# Customs of Russian Doctors

**Published:** 1896-12

**Authors:** 


### Customs of Russian Doctors.

The Russian physician, the *Record* says, considers it beneath his dignity to send a bill to a patient, but leaves it to the patient to pay what he thinks proper. Many think it proper to pay nothing. Are you acting the Russian in business matters? If so, neither free silver, free gold, nor anything can save you from financial distress.—*Kansas City Medical Index.*

